# Pulmonary Injury, Functional Impairment, and Transcriptomic Responses Following Phosphine Inhalation in Mice: Implications for Therapeutic Development

**DOI:** 10.1021/acs.chemrestox.6c00223

**Published:** 2026-09-28

**Authors:** Esraa Mohamed, Andrea Adamcakova-Dodd, Xuefang Jing, David K. Meyerholz, Mohammad Al-Natour, Peter S. Thorne, Aliasger K. Salem

**Affiliations:** † Department of Pharmaceutical Sciences and Experimental Therapeutics, College of Pharmacy, 4083 University of Iowa, Iowa City, Iowa 52242, United States; ‡ Occupational and Environmental Health, College of Public Health, 50692 University of Iowa, Iowa City, Iowa 52242, United States; § Department of Pathology, Carver College of Medicine, University of Iowa, Iowa City, Iowa 52242, United States

## Abstract

Phosphine (PH_3_) is a highly lethal gas that is widely used as an agricultural fumigant. Human exposure occurs through inhalation of accidentally released gas or phosphide-containing pesticides. Its high lethality, combined with its ready accessibility, makes it a chemical threat agent. Although its toxic mechanism remains unclear, PH_3_ is known to induce oxidative stress, leading to cell death. With no available antidote, this study examines the pulmonary effects of acute PH_3_ inhalation exposure in order to support future treatment strategies and identifies key biological pathways affected in the lungs and hearts. Female mice were exposed acutely to one of three PH_3_ doses in a dynamic whole-body exposure chamber. Following exposure, respiratory function, total protein levels in bronchoalveolar lavage fluid (BALF), and lung pathology were evaluated. Bulk RNA sequencing of PH_3_-exposed lungs and hearts was performed, and differentially expressed genes (DEGs) in tissues from exposed versus nonexposed mice were analyzed. Animals exposed to high PH_3_ doses experienced a significant decrease in respiratory rate and had to be euthanized within 5 h postexposure. There was a significant increase in total protein and cell counts in BALF of PH_3_-exposed mice at the medium and high doses, and histopathological evaluation of lungs showed foci of hypercellularity and scattered vessels filled with platelet aggregates and neutrophils, indicative of early pulmonary thrombi formation. Gene expression analysis revealed upregulation of genes involved in protein synthesis, inflammation, cell cycle, and apoptosis. The MAPK, IL-17, and atherosclerosis pathways were among the most impacted pathways in lung and heart tissues. *Fos, Fosb, Jun*, and *Junb* were significantly upregulated following exposure; all of them are components of the AP-1 protein complex, a potentially key element in inducing injuries subsequent to PH_3_ exposure and therefore a promising therapeutic target.

## Graphical abstract

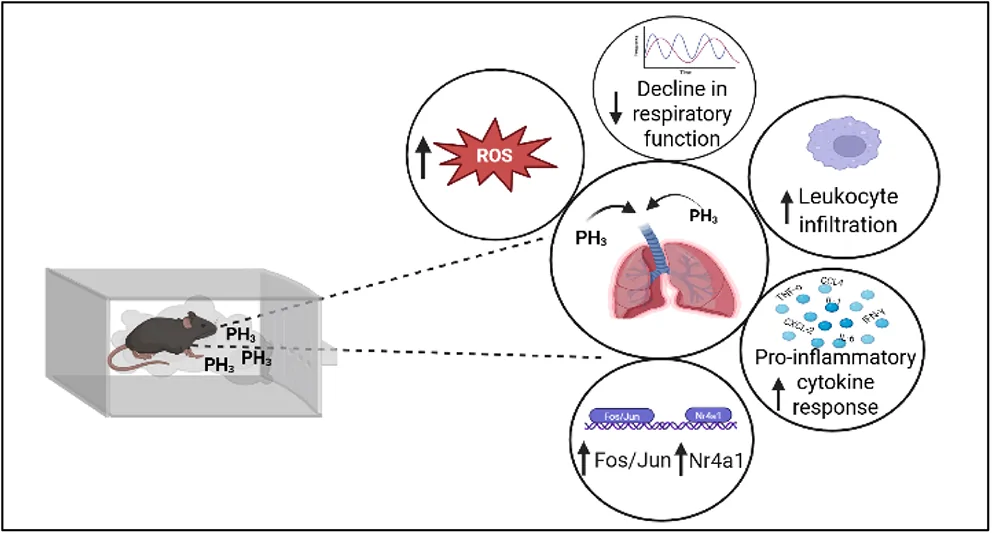

## 1 Introduction

A variety of pesticides are routinely applied worldwide to enhance the quality of agricultural products.^1^ Despite the important role of pesticides in saving crops, they pose a significant threat to human and animal health if used improperly.^2^ The World Health Organization (WHO) estimated that approximately one million unintentional pesticide poisonings occur annually, leading to approximately 20,000 deaths.^3^ One of the most commonly used fumigants that effectively eliminate pests and other organisms that can infest cargo during transportation is phosphine (PH_3_). PH_3_ is a colorless, flammable, and highly fatal gas that has a fishy or garlic-like odor; however, it is odorless in its pure form.^4^ The odor threshold at which humans can detect PH_3_ gas does not provide sufficient warning of dangerous concentrations, which may lead to unintentional exposure to high concentrations.^1,5^ PH_3_ is one of the most commonly used fumigants because of its effectiveness against diverse pest species, low application cost, and lack of harmful residues on treated cargo.^6–9^ In addition, PH_3_ has a wide industrial use (e.g., in the semiconductor industry;^10^ as a polymerization initiator; in preparation of flame retardants; and as a condensation catalyst^11,12^ ). The OSHA permissible exposure limit (PEL) for PH_3_ is 0.3 ppm as an 8-h time-weighted average, with a short-term exposure limit of 1 ppm for 15 min.^5^ The US Environmental Protection Agency has categorized PH_3_ or its phosphide form, aluminum phosphide (AlP), in Toxicity Category I, which is the most toxic of the four categories.^13,14^

Human exposure to PH_3_ occurs by inhalation of accidentally released gas, or through intentional ingestion of phosphide-containing pesticides (metal phosphides), in which case phosphides come into contact with water or hydrochloric acid in the stomach, releasing PH_3_ gas.^1,15,16^ Due to the widespread use and availability of metal phosphides and the devastating health consequences associated with exposure, the use of AlP and zinc phosphide for suicide or homicide especially in developing countries has become common especially among adolescents.^8,16–18^ PH_3_ poisoning represents one of the most common causes of poisoning-related fatalities in India,^15,19^ Egypt,^18^ Iran,^10,17^ Morocco, and many other countries.

The symptoms of PH_3_ toxicity are nonspecific, and the severity can depend on the level of exposure.^2^ Exposure to PH_3_ can cause toxicity related to the respiratory, cardiovascular,^20^ central nervous,^21^ and gastrointestinal systems.^8^ Pulmonary symptoms include lung irritation, chest tightness, cough, shortness of breath, and pulmonary edema.^1,12,16^ Cardiovascular effects include abnormally low blood pressure, irregular heart rhythm, and low ejection fraction.^10^ Nervous system effects include headache, restlessness, irritability, dizziness, lethargy, trembling of the extremities, and double vision.^11^ Gastrointestinal effects include nausea, vomiting, diarrhea, abdominal pain,^22^ gastrointestinal distress,^5^ as well as kidney and liver damage.^23^ The adverse health effects generally occur within the first few hours following inhalation of PH_3_ and have a high mortality rate ranging from 30% to more than 80%.^2,9,22,24,25^

The mechanism of toxicity caused by PH_3_ is not fully understood,^7^ although the following mechanisms have been suggested: cellular hypoxia due to inhibition of the mitochondrial complexes; inhibition of cytochrome c oxidase; and formation of highly reactive hydroxyl radicals that cause cellular injury due to lipid peroxidation.^2,4,7,26,27^ Previous studies showed that there was a reduction in the levels of catalase, superoxide dismutase, and glutathione peroxidase in different tissues after AlP poisoning.^16,20^ Until now, most studies have investigated PH_3_ toxicity after ingestion,^4,28^ while far fewer studies have focused on adverse effects after inhalation exposure,^29–31^ and thus there is a need for further investigation of this exposure route.

Despite the increased frequency of PH_3_ poisoning with high fatality rates (within the first 24 h after exposure), there is no available antidote.^16^ Treatment is usually supportive^9^ (e.g., removal from the exposure source; supply of humidified oxygen; respiratory and cardiac function evaluation;^23^ and control of symptoms and prevention of complications by administration of antioxidants^16^ such as N-acetyl cysteine).^2,19^ High fatality rates within the first few hours of exposure are attributed to cardiac toxicity and respiratory arrest;^32^ however, the molecular mechanisms underlying these toxicities remain incompletely understood. Therefore, there is a critical need to define reliable therapeutic targets and find effective therapies that can mitigate the detrimental effects of PH_3_ exposure on heart and lung tissues.

The aim of our study was to evaluate respiratory function and pulmonary toxicity, as the lung represents the first and most directly exposed target organ after inhalational exposure, and to identify genomic changes and key molecular pathways in lungs and hearts in order to understand the mechanism of PH_3_-induced toxicity after inhalation exposure. Our study represents a comprehensive analysis of potential molecular mechanisms underlying lung injury following PH_3_ exposure, and these may offer insights into molecular mechanisms associated with PH_3_ injuries to the heart and other organs. Such identified pathways can then serve as possible targets for treatment strategies.

## 2 Materials and Methods

### 2.1 Animals

All experiments were performed using C57BI/6J, 5-week-old female mice purchased from The Jackson Laboratories (Bar Harbor, ME). Female mice were selected to study a potentially more vulnerable population. Additionally, previous work evaluating sex as a biological variable in PH_3_ inhalation toxicity exhibited comparable lethality and overall cardiopulmonary responses, with minor sex-related differences observed in the timing of certain physiological responses.^33^ However, future studies using both sexes are necessary to determine whether similar responses occur in male mice. Animals were housed in an AAALAC-accredited vivarium, in polypropylene, filter-covered cages in HEPA-filtered Thoren caging units (Hazleton, PA). Animals were acclimatized for 7 days after their arrival and were maintained at standard temperature and humidity, in a 12 h dark/light cycle under conditions with standard diet and water available *ad libitum.* All animal experiments were performed following the protocols evaluated and approved by the Institutional Animal Care and Use Committee (IACUC, Animal Protocol Approval Number: 3091097).^34^ The first experiments performed were conducted under close surveillance by a veterinarian.

### 2.2 Mouse Exposure to PH_3_ Gas

The system for the generation of PH_3_ gas as well as a dynamic whole-body exposure chamber was placed in a full-height chemical hood and inspected by the University of Iowa Environmental Health and Safety personnel prior to the start of the exposures. PH_3_ gas was generated from a compressed gas cylinder (984 ppm of PH_3_ balanced with nitrogen, 2200 psi, Linde, Danbury, CT), equipped with pressure and flow regulators, and was diluted with HEPA-filtered lab air using mass flow controllers (Alicat Scientific, Inc., Tucson, AZ). During exposure, both PH_3_ and O_2_ levels were monitored continuously to make sure that the O_2_ concentration inside the chamber did not decrease below 13%. O_2_ was supplied from O_2_ concentrator. The atmosphere outside the chemical hood was continuously monitored for PH_3_ concentration (MX6, iBRID, Industrial Scientific, Pittsburgh, PA) that was set to alarm at 3 ppm. Mice were exposed in the dynamic whole-body exposure chamber under precisely controlled conditions (Figure 1) to one of three doses of PH_3_ gas: 12,000 ppm × min, 16,000 ppm × min and 20,000–22,500 ppm × min that represent low, medium, and high PH_3_ doses, respectively.^29^ Additional lower doses (2,000–7,200 ppm × min) were initially evaluated based on preliminary *in vitro* findings; however, they did not produce observable signs of toxicity in mice (data not shown). Immediately after exposure, mice were administered an analgesic (2 mg/kg, Buprenorphine SR) by subcutaneous injection to minimize pain and distress after exposure and the mice were closely monitored. Control animals not exposed to PH_3_ received the same dose of analgesic as the PH_3_-exposed mice. All mice were carefully observed for clinical signs of PH_3_ toxicity, and the number of surviving and deceased mice was recorded. These survival data were used to determine the lethality range of PH_3_ exposure. Respiratory parameters were recorded in animals at multiple time points. Mice were euthanized (by an overdose of isoflurane followed by cervical dislocation and cardiac exsanguination) at various intervals (0.5–96 h) after exposure, as demonstrated in Table 1. BALF (from right lung lobes), lungs, and hearts were collected. All specimen (except right lung lobes that were fixed in formalin) were first frozen in liquid nitrogen and then stored at −80 °C until analysis or stored in RNA-later buffer as specified below.

**Figure 1:**
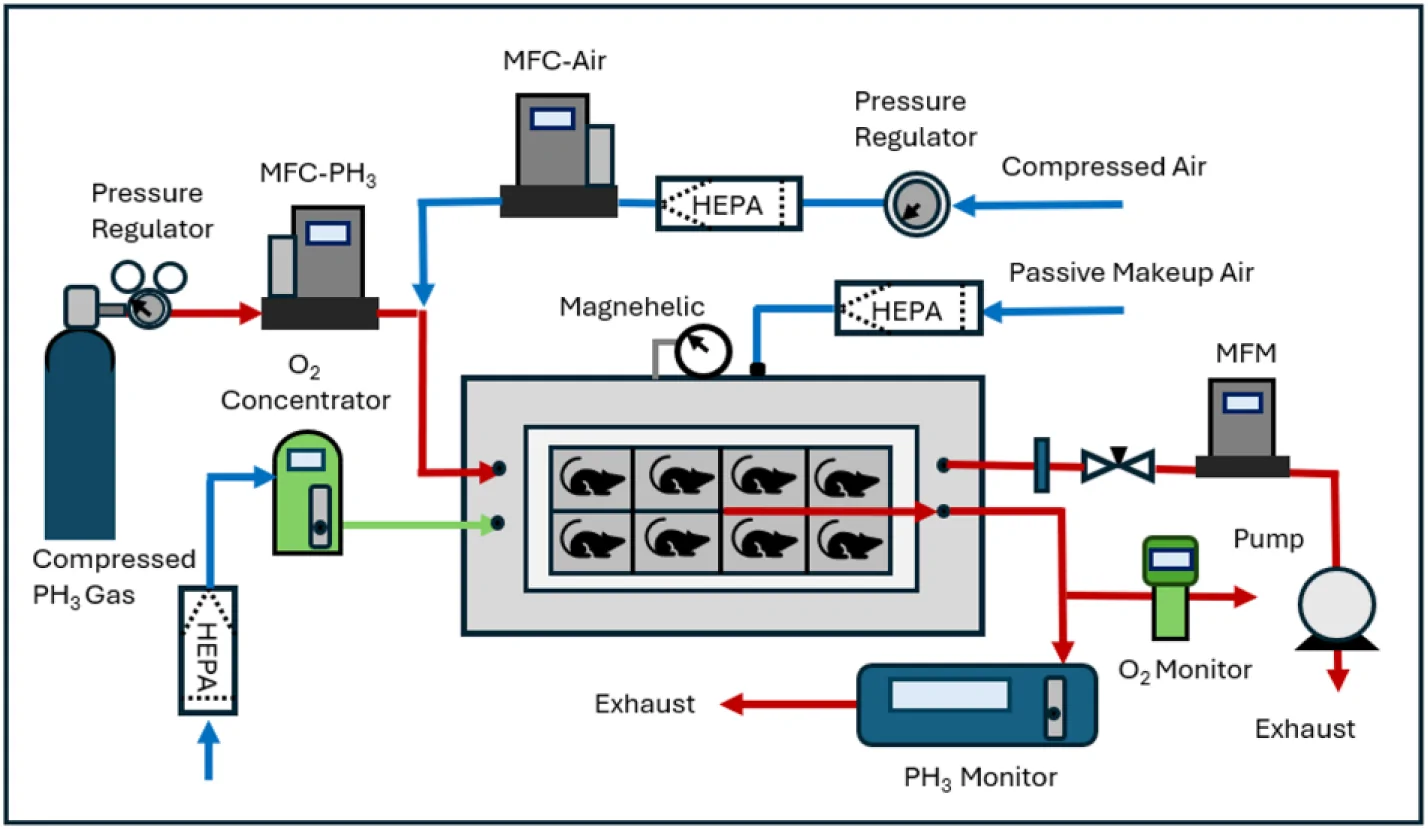
Schematic illustrating the system setup for exposure of animals to PH_3_ gas. HEPA: High-Efficiency Particulate Air filter, MFC: Mass Flow Controller, MFM: Mass Flow Meter.

**Table 1:** Experimental Groups, Exposure Concentrations, Necropsy Time Points, and Number of Animals per Group

Group ID	PH_3_ gas Concentration (ppm)	Duration (min)	Concentration × time (ppm × min)	Necropsy time after exposure (h)	*n* per group
Control	0	0	0	24	6
Low dose	600	20	12,000	96	5
Medium dose_N1	500	32	16,000	24	4
Medium dose_N2	500	32	16,000	96	5
High1 dose	500	40	20,000	2–5	4
High2 dose	900	25	22,500	0.5–4	6

### 2.3 Clinical Observations

During and after exposure, all mice were closely observed for clinical signs of PH_3_ toxicity. Behavioral, physical, and physiological changes using the Rodent Grimace Scale (orbital tightening, nose/cheek bulge, ear position) were monitored, along with hunched posture, reduced grooming (rough coat), decreased activity, excitability, lethargy, and ataxia.^31^

### 2.4 Respiratory Function Assessment

Whole-body plethysmography (WBP) was used to monitor tidal volume, respiratory airflow, and respiratory effort in unrestrained, freely moving mice (Emka Technologies).^35,36^ Mice were placed in the WBP chambers, and respiratory recordings were obtained simultaneously during multiple 10-s periods over 30 min using IOX acquisition software (Version IOX 2.10.5.14, Emka Technologies, Canada). Respiratory functions were measured before the exposure (baseline) and at several time points postexposure (0, 2, 4, 24, 48, and 96 h). The respiratory functions of unexposed mice were recorded at each time point and used as controls for comparisons. Breathing frequency was expressed as breaths per minute (bpm). Absolute values for inspiratory and expiratory time (mSec), minute volume (mL), and inspiratory and expiratory flow rates (mL/sec) were used.

### 2.5 BALF Analysis

After euthanasia, right lobes of the lungs were lavaged three times with 1 mL of 0.9% (w/v) sterile sodium chloride solution (Baxter, Bloomington, Indiana). The retrieved BALF was centrifuged at 800 × *g* for 5 min; the cell pellet was then used for total and differential cell count determination as previously described.^37^ The cell pellets were resuspended in 200 μL Hank’s balanced salt solution (HBSS, Life Technologies, Carlsbad, CA). Both cell viability using propidium iodide staining and total cell counts were measured using MFS cassettes (MXC020) and an automated cell counter (Moxi GO II, Orflo Technologies, Ketchum, ID). To determine the differential cell counts including macrophages, neutrophils, lymphocytes, and eosinophils, cells were spun (Cytospin 4, Thermo Scientific) and fixed on glass microscope slides with fetal calf serum. Staining was performed using Protocol HEMA 3 stain set (Fisher Scientific, Hampton, NH), and 400 total cells were blindly evaluated under an optical microscope (Olympus, Tokyo, Japan) to differentiate and count the cell types.

The BALF supernatants were used for analyses of total protein using the Micro BCA Protein Assay Kit (Thermo Fisher Scientific, Rockford, IL). Samples and standards were incubated with the BCA working reagent for 2 h at 37 °C. After incubation, the absorbance of standards and samples was measured at 562 nm using a SpectraMax M5 microplate reader (Molecular Devices, San Jose, CA). A standard curve with bovine serum albumin was generated to determine protein concentrations of the samples.

### 2.6 Histopathological Examinations

The right lobes of the lungs after BALF collection were first perfused and preserved in 10% neutral-buffered formalin (1–2 weeks) until processing. The tissues were then paraffin-embedded, sectioned (5 μm thickness), and stained with hematoxylin and eosin (H&E).^34,38^ The sections were evaluated using brightfield light microscopy (BX 53 microscope, Olympus, Center Valley, PA) and high-resolution digital imaging (DP73 digital camera and cellSens Dimension software, Olympus). Histopathological examination was performed by a board-certified veterinary pathologist using a port-examination method of masking to prevent bias and following the principles of reproducible tissue analysis.^39,40^

### 2.7 Human Bronchial Epithelial Cell Exposure to PH_3_ Gas and Oxidative Stress

Human bronchial epithelial cells (HBECs, ATCC, Manassas, VA) were cultured in airway epithelial cell growth medium (ATCC) at 37 °C in a humidified atmosphere containing 5% CO_2_ until reaching 80–85% confluency. The cells were dissociated with 0.05% trypsin-0.02% EDTA (ATCC) and counted using a hemocytometer. Cells were seeded onto the apical side of Transwell membrane inserts (polyester, 12 mm diameter, 0.4 μm pore size, Corning, USA) at a density of 1.25 × 10^5^ cells/mL. Cells were grown for 24 h under submerged conditions, then the apical medium was removed, and cells were incubated for an additional 14–16 h under air–liquid interface conditions. Cells were placed inside a 2 L-glass exposure chamber where they were exposed to PH_3_ gas (50, 150, or 300 ppm for 10 min with total exposure concentration of 500, 1500, or 3000 ppm × min, respectively).

Intracellular reactive oxygen species (ROS) were assessed using chloromethyl-2′,7′-dichlorodihydrofluorescein diacetate (CM-H2DCFDA, Thermo Fisher Scientific). CM-H2DCFDA passively penetrates cells and reacts with ROS to form the highly fluorescent 2′,7′-dichlorofluorescein (DCF).^4,41^ Higher DCF fluorescence intensity after exposure means higher levels of ROS generated by the cells. Cells on Transwell inserts were rinsed twice with 250 μL of PBS; then, 500 μL of HBSS (with Ca^2+^ and Mg^2+^) containing 25 μM CM-H2DCFDA was added to the apical side of the cells and incubated in the dark for 30 min at 37 °C, then the HBSS solution was removed, and cells were washed again. After washing, cells were lysed with 0.1% Triton X-100 in PBS for 30 min at 37 °C, and all lysates were collected into black microcentrifuge tubes and centrifuged at 16,000 × *g* for 15 min at 4 °C. Supernatant fluorescence intensities were measured with excitation at 494 nm and emission at 522 nm (SpectraMax M5).

The cell viability after PH_3_ exposure was evaluated using the alamarBlue Cell Viability Reagent (Thermo Fisher Scientific). Cells on Transwell inserts were rinsed twice with PBS and then incubated with the alamarBlue Cell Viability Reagent for 2 h. The increased fluorescence was detected at 560 nm excitation and 590 nm emission wavelength. Cells not exposed to PH_3_ were used as a control for cell viability percentage calculation. The relative mean fluorescence intensity (RMFI) of the DCF compound was normalized to the percentage cell viability postexposure.

### 2.8 RNA Sequencing

Lung tissue not used for recovery of BALF and heart tissues were harvested, cut into small pieces of 0.5 cm thickness, and stored in RNAlater Solution (Thermo Fisher Scientific) at −20 °C until further processing was initiated. For RNA isolation, tissues were retrieved from RNAlater Solution, with excess solution being removed with a lab wipe, and then submerged in RNA isolation lysis solution containing β-mercaptoethanol. The sample was homogenized using a Tissue Ruptor II (QIAGEN, Hilden, Germany) on ice until the lysate was homogeneous. The homogenate was centrifuged at 12,000*g* for 3 min at 15–25 °C. The supernatant was aliquoted to RNeasy Mini spin columns, following the QIAGEN RNeasy Fibrous Tissue Mini Kit (QIAGEN) protocol with DNase I digestion on the column to clean and isolate RNA. RNA was eluted using RNase-free water. Purity and concentrations were quantified using a NanoDrop spectrophotometer (Thermo Fisher Scientific), and samples were stored at −80 °C. All samples that met the RNA quality requirements (RNA concentration >20 ng/μL, 260/280 ratio >2, and 260/230 ratio >1.9) were shipped to Novogene (Davis, California, USA) for library construction and Illumina RNA sequencing. The integrity of the RNA samples was evaluated by Novogene, the average RNA Integrity Number (RIN) for all evaluated samples was 9.3 ± 0.4 (Table S1).

The effects of PH_3_ were evaluated by comparing gene expression in each exposure group (medium and high2) to that of the controls (n = 3 per group). Only one high-dose group was selected for transcriptomic analysis because high1 and high2 produced similar respiratory, survival, and histopathological outcomes. To compare molecular changes between nonlethal (medium dose; 0% mortality) and lethal exposure (100% mortality), we selected the high2 group for RNA sequencing, as it represented the highest concentration tested while yielding outcomes comparable to high1. Differentially expressed genes were analyzed to identify biological pathways and disease ontologies affected by PH_3_ inhalation using a threshold of 0.05 for statistical significance (padj) and a log 2-fold change of expression with an absolute value of at least 1. The clusterProfiler software was used for enrichment analysis, including gene ontology (GO) enrichment and the Kyoto Encyclopedia of Genes and Genomes (KEGG) pathway analysis to determine which biological functions or pathways were significantly associated with the differentially expressed genes.

### 2.9 Statistical Analysis

All data values were reported as mean ± SD, except for the WBP data that were reported as mean ± SE. Statistical significance was calculated with one-way ANOVA, followed by Dunnett’s posthoc test if more than two groups were compared. Unpaired two-tailed Student’s *t*-test was utilized when comparing two experimental groups. *p*-values of less than or equal to 0.05 were considered significant. Differential expression analysis was performed on gene-level RNA-seq count data using R and DESeq2 using a threshold of 0.05 for statistical significance (padj) and a log 2-fold change of expression with absolute value of at least 1. GO and KEGG terms with padj <0.05 were considered significant.

## 3 Results and Discussion

### 3.1 Mouse Exposure to PH_3_ Gas

Female mice (C57BI/6J, 5 weeks old) were exposed to low, medium, and two high PH_3_ doses. The target concentrations of O_2_ and PH_3_ were achieved in the exposure chamber within the first 2–3 min and maintained during the exposure period (Figure S1). Table 1 shows the experimental groups and the number of animals per group. While the sample sizes for each experimental group were lower (4–6 animals/group), we performed a power analysis using the BALF total protein level as one of the end points representing lung injury, and the results showed that a sample size of 4–6 mice per group provided 95% statistical power with an α level of 0.05. In addition, the consistency of the observed results across measured end points supports the biological relevance of the reported effects. Future studies with a larger cohort will be valuable to confirm our findings and increase statistical robustness. Necropsies were conducted at different time points post-exposure based on the clinical observation of mice. The survival analysis (Figure 2) revealed that the lethality range for PH_3_ was between 16,000 and 20,000 ppm × min, corresponding to doses that resulted in complete survival or complete mortality, respectively.

**Figure 2:**
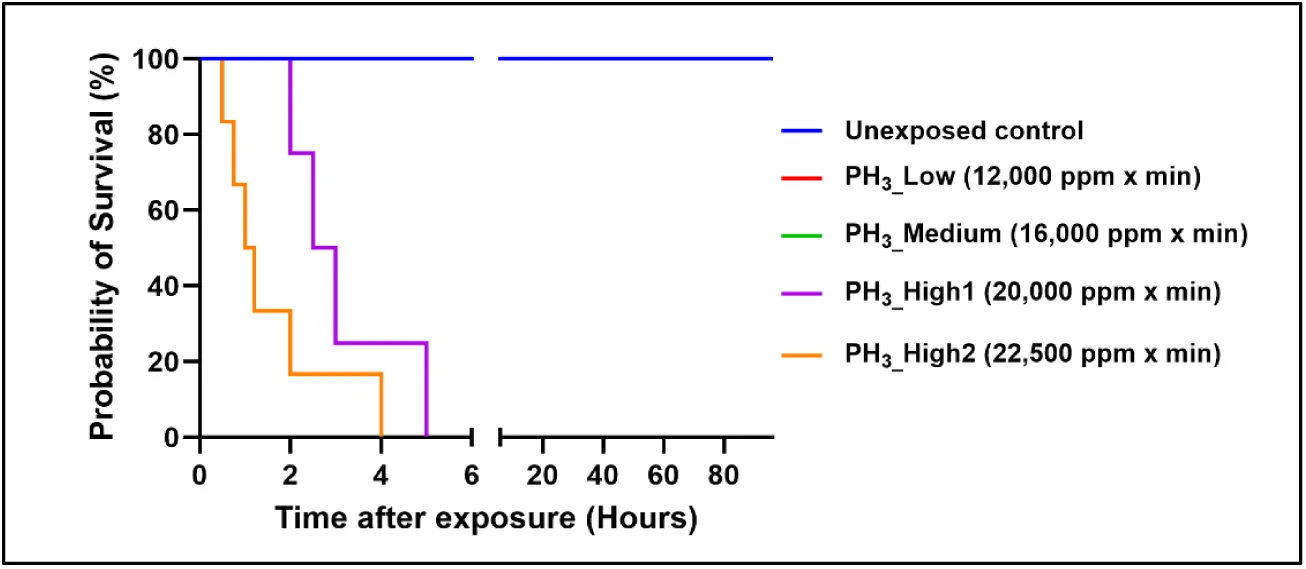
Kaplan–Meier estimate of survival of animals exposed to different concentrations of PH_3_ gas compared to the control group (*n* = 4–6 per group). The groups exposed to low and medium PH_3_ doses and the control group show overlapping survival probabilities.

### 3.2 Clinical Observations

All mice were closely monitored immediately after exposure and hourly up to 6 h postexposure to ensure the absence of severe injuries that could affect their survival. After that, mice were observed at 12, 24, 48, 72, and 96 h postexposure. Groups exposed to low and medium PH_3_ doses were active immediately after exposure at the initial time points (0.5, 1, 2, 3, 4, 5, and 6 h), did not exhibit significant adverse effects, and survived until the scheduled necropsy time points (24 or 96 h). Conversely, we observed a distinct clinical effect with high PH_3_ doses, characterized by morbidity (piloerection, hunching, decreased respiratory rate, and dyspnea), leading to a severe clinical threshold requiring euthanasia per IACUC guidelines within 0.5–5 h postexposure (Figure 2). These observations highlight the pronounced lethal effect of higher PH_3_ doses and suggest the potential for tissue and systemic damage following exposure.

### 3.3 Respiratory Function Assessment

Respiratory functions of mice exposed to varying doses of PH_3_ were assessed at multiple time points postexposure (0, 0.5, 2, 4, 24, 48, and 96 h) and compared to the control group without exposure to the gas. There were significant alterations in respiratory frequency, breathing pattern, and ventilation after PH_3_ gas exposure. Mice exposed to medium and high PH_3_ doses exhibited a pronounced decrease in respiratory rate, minute volume, peak inspiratory and expiratory flow rates, and an increase in inspiratory and expiratory times (Figure 3). In addition, there was a notable increase in the enhanced pause (*P*_enh_) following PH_3_ exposure. These findings indicate profound sensory and pulmonary irritation. The significant changes observed after medium-dose exposure lasted for the early hours after exposure and started showing a normal pattern similar to the control group by 24 h following exposure.

**Figure 3:**
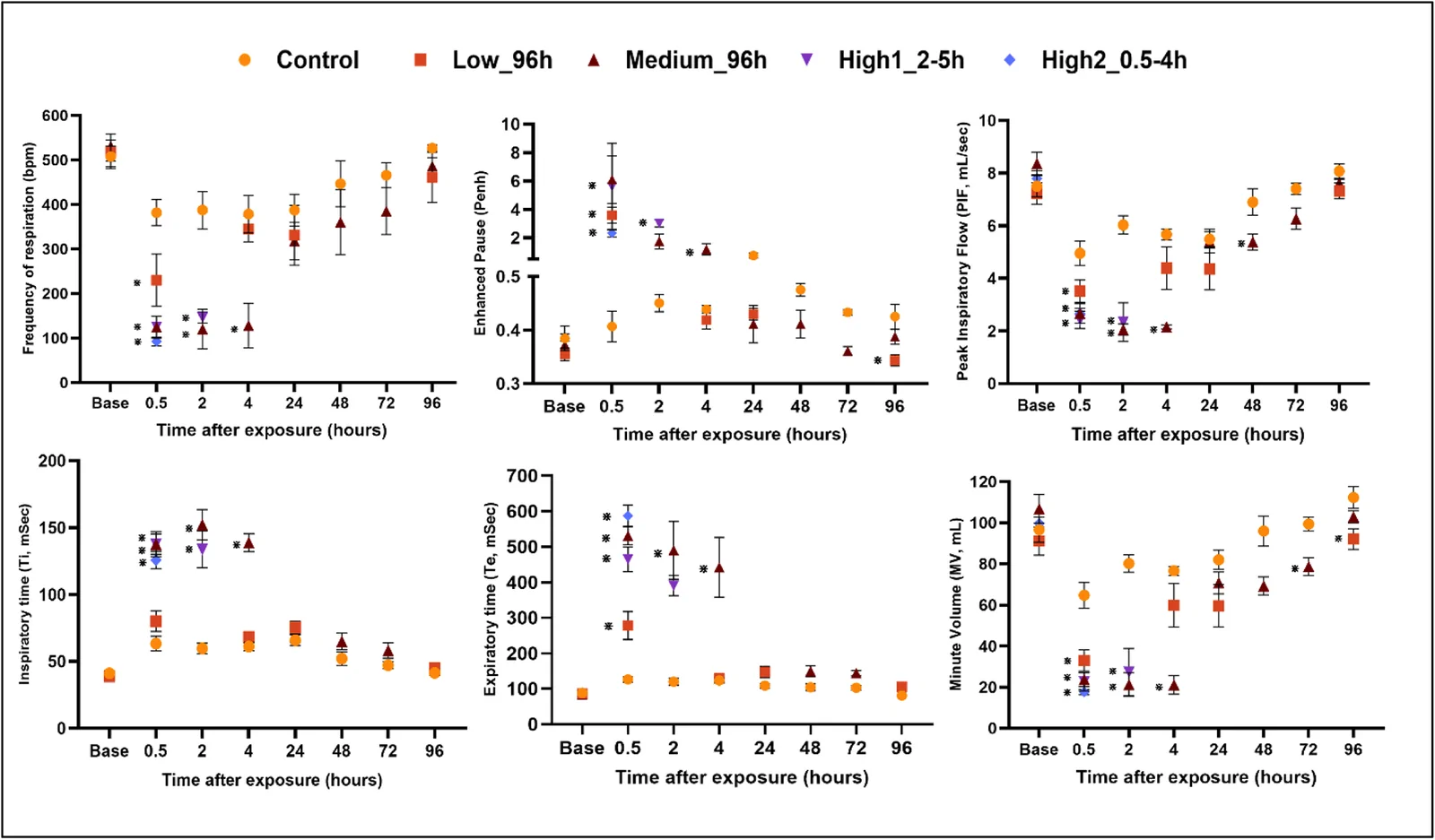
Respiratory function parameters measured by whole-body plethysmography at several time points post-exposure to PH_3_ gas (*n* = 4–6 per group). Values represent mean ± SE. Statistical analysis was performed using one-way ANOVA followed by Dunnett’s posthoc test. “
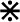
” represents the statistical difference between groups and control group at each time point with *p* < 0.05.

### 3.4 Total Protein Levels in BALF

The blood-air barrier can be disrupted in response to gas exposure, causing an influx of fluid and proteins into the alveolar space. The BALF of groups exposed to Medium and High doses showed a significant increase in total protein concentration compared to the unexposed control, up to 24 h postexposure, as shown in Figure 4A. Protein levels returned to normal levels (comparable to unexposed mice) within 96 h for the group exposed to medium doses. These findings aligned with the WBP results, which showed no persistent changes in respiratory parameters compared to controls at 96 h postexposure. The normalization of the total protein level, together with the return of the respiratory parameters toward baseline values at 96 h postexposure, suggests that there was functional restoration after acute lung injury at the medium dose. This response may involve cellular pathways and modulation of immune responses; however, further investigation is needed to confirm the specific cellular and molecular pathways involved.

**Figure 4:**
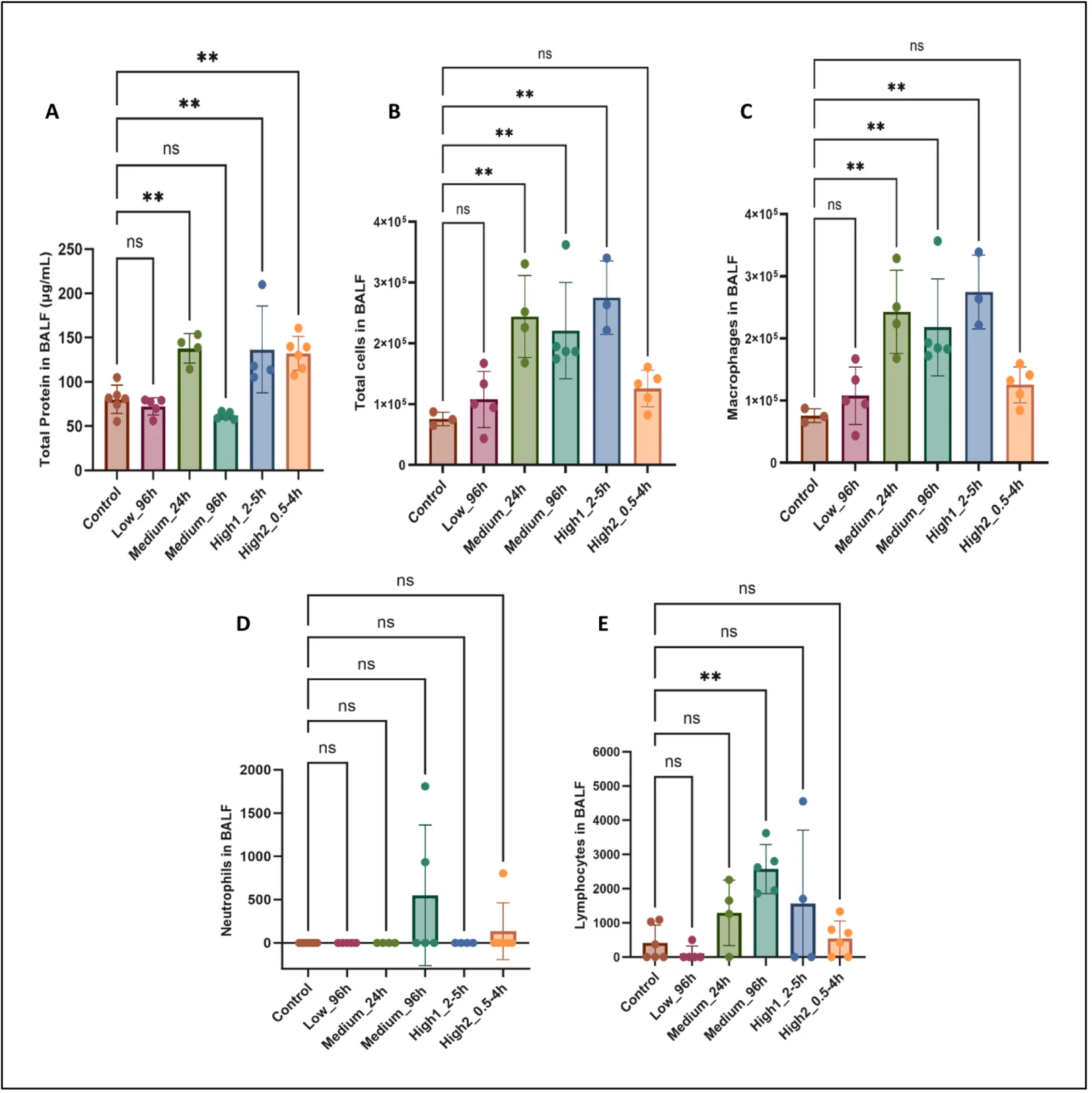
Total protein concentration (A) and total cell count (B) and number of macrophages (C), neutrophils (D), and lymphocytes (E) per mouse in the BALF of mice treated with the indicated PH_3_ gas concentrations (*n* = 4–6 per group). Values represent means ± SD. Statistical analysis was performed using one-way ANOVA followed by Dunnett’s posthoc test. *p < 0.05, ** *p* < 0.01, ***p < 0.001, ****p < 0.0001.

### 3.5 Total and Differential Cell Counts in BALF

Cellular composition in the BALF showed that inflammatory cell counts, including macrophages, were higher in the groups exposed to medium and high doses of PH_3_ compared to the unexposed controls (Figure 4B–E). However, neutrophil and lymphocyte counts were comparable between exposed and unexposed groups at 24 h postexposure. No eosinophils were found in the BALF. The low count of neutrophils and lymphocytes suggests the absence of an acute inflammatory response shortly after exposure. However, the lymphocyte count increased significantly 96 h after exposure. This suggests that the observed changes in the cellular composition postexposure to PH_3_ gas could be related to delayed rather than acute inflammatory responses.

### 3.6 Histopathological Evaluation

Histopathological evaluations of lung tissues from mice exposed to different concentrations of PH_3_ revealed that there were minimal changes between the lung tissues exposed to a medium dose of PH_3_ at 24 or 96 h postexposure compared to the control group, with both groups demonstrating a few regions of hypercellularity near the blood vessels that were mostly mononuclear, with a few mildly hypercellular septa in the group euthanized 24 h postexposure. However, the groups exposed to high doses showed regions with multifocal to coalescing hypercellularity of septa and multiple vessels filled with platelet aggregates and neutrophils. These aggregates were early signs of thrombus formation. Pulmonary thrombi can form because of local or systemic lung inflammation, and their early appearance is often characterized by platelet aggregates (platelet thrombi) with neutrophils. Although neutrophils were not detected in the BALF of the exposed groups, there were pronounced neutrophilic aggregates associated with vascular thrombi in the lung tissues of the groups exposed to higher PH_3_ concentrations (Figure 5). This suggests that, after PH_3_ exposure, neutrophils may begin to localize within the pulmonary tissues and vascular compartment rather than migrating into the air space. This emphasizes that BALF measurements alone may underestimate pulmonary inflammation following exposure. It is possible that the acute pulmonary thrombi in the high-dose groups contributed to clinical morbidity and clinical decline, necessitating euthanasia. These findings are consistent with the observed deterioration in pulmonary function, increased cellular infiltration, and elevated protein concentration detected in the BALF at high PH_3_ doses.

**Figure 5:**
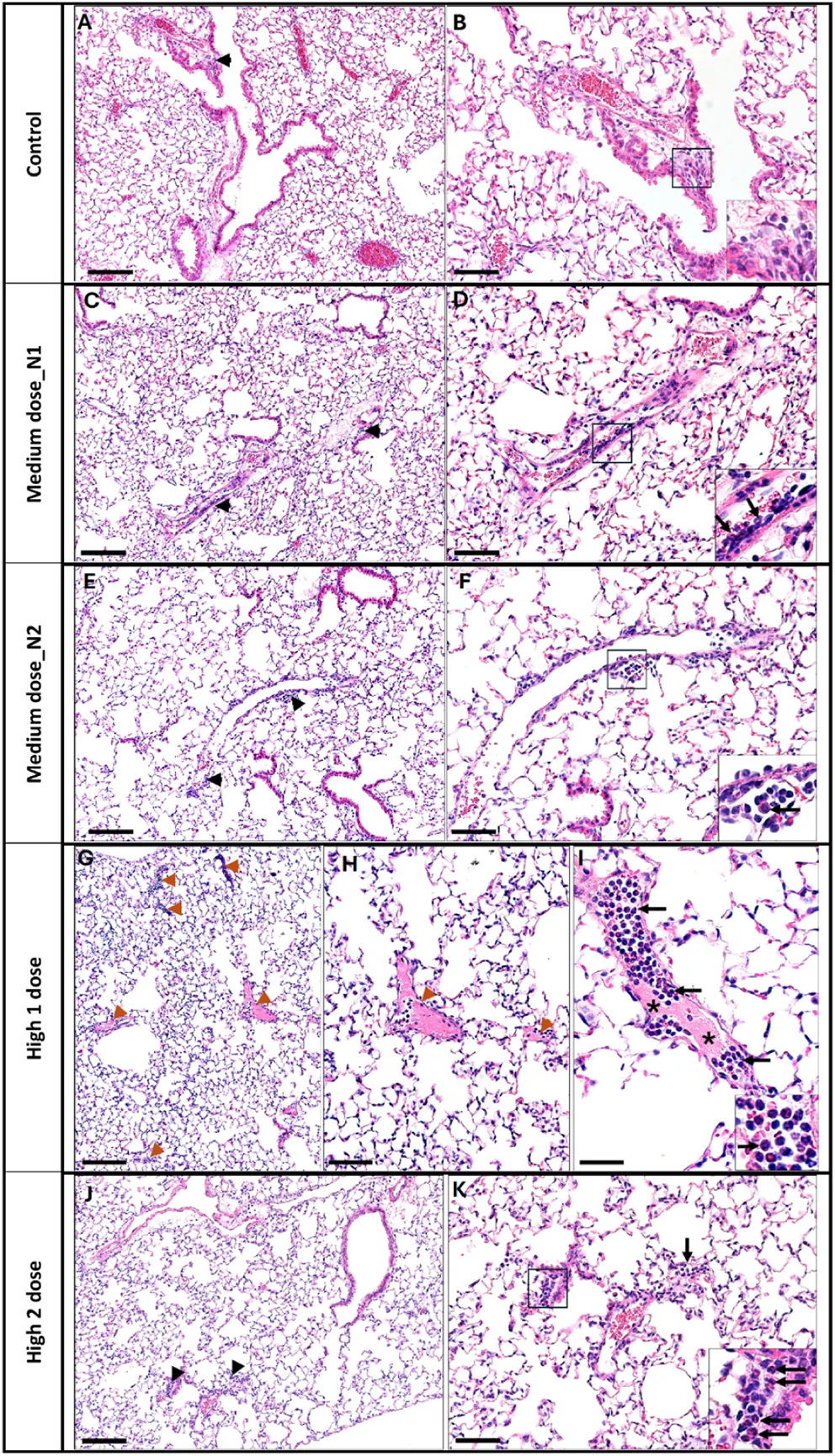
Histopathology of lungs from control (unexposed) group and groups exposed to medium and high PH_3_ doses. The images show regions of hypercellularity (black arrowheads) and multiple vessels (orange arrowheads) variably filled with a mix of platelet aggregates (*) and neutrophils (arrows and inset). Scale bars represent 133 μm for A, C, E, and J and 66 μm for B, D, F, and K. For high1 dose, the scale bars represent 147 μm for G, 73 μm for H, and 36 μm for I.

### 3.7 In Vitro Oxidative Stress Assay

Human bronchial epithelial cells (HBECs) were exposed to either room air or different doses of PH_3_ gas for 10 min. At different time points postexposure, intracellular ROS levels and cell viability were assessed (Figure S2). A significant dose-dependent increase in ROS levels was found in response to PH_3_ exposure (Figure 6). The RMFI was higher in the groups exposed to 500, 1500, or 3000 ppm × min up to 24 h after exposure compared to the unexposed control. These findings suggest that PH_3_ gas exerts its toxicity through the generation of highly reactive free radicals. This conclusion aligns with previous studies that have shown a relation between ROS, mitochondrial dysfunction, and detrimental effects following PH_3_ gas exposure.^8^

**Figure 6:**
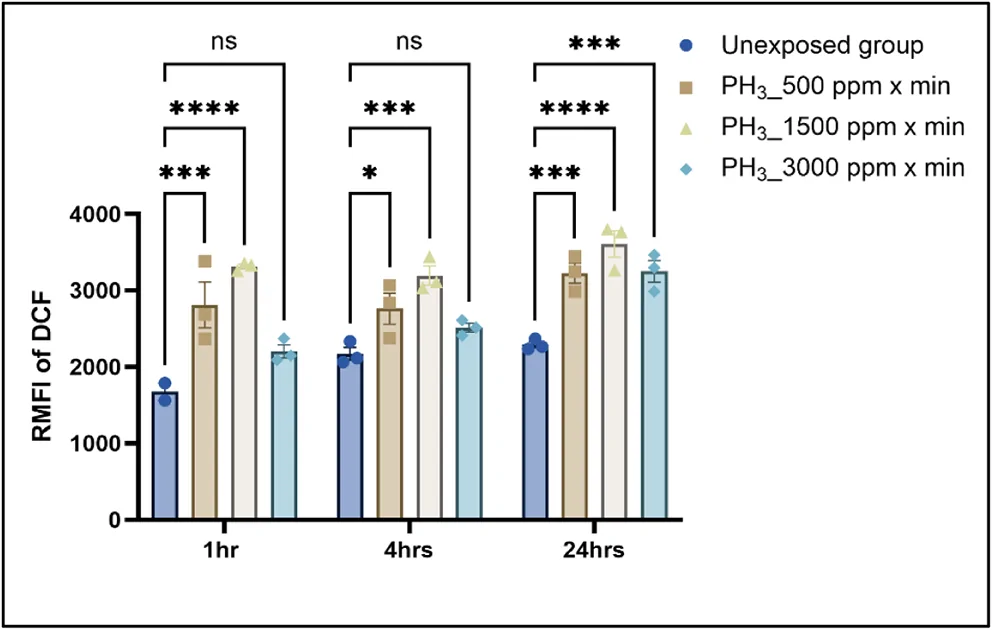
Intracellular ROS in HBECs measured as DCF relative mean fluorescence intensity (RMFI: normalized to cell viability assessed using alamarBlue) after exposure to different PH_3_ gas concentrations compared to unexposed cells. The evaluation was performed at 1, 4, and 24 h following exposure (values represent means ± SD). Statistical analysis was performed using two-way ANOVA. *p < 0.05, ** *p* < 0.01, ***p < 0.001, ****p < 0.0001.

### 3.8 mRNA Sequencing Analysis

We analyzed changes in the mouse lung transcriptome following PH_3_ exposure using mRNA sequencing (RNAseq) analysis in an attempt to understand the mechanism(s) underlying lung damage and altered pulmonary function after PH_3_ exposure. Additionally, we assessed transcriptomic changes in the heart following exposure to determine whether respiratory injuries were regulated by pathways distinct from those in cardiac tissue. For pathway analyses, each exposure group was compared with the controls. The principal component analysis (PCA) of gene expression levels demonstrated an obvious distinction between the exposed and control groups and confirmed the reproducibility of the samples within each group (Figure 7).

**Figure 7:**
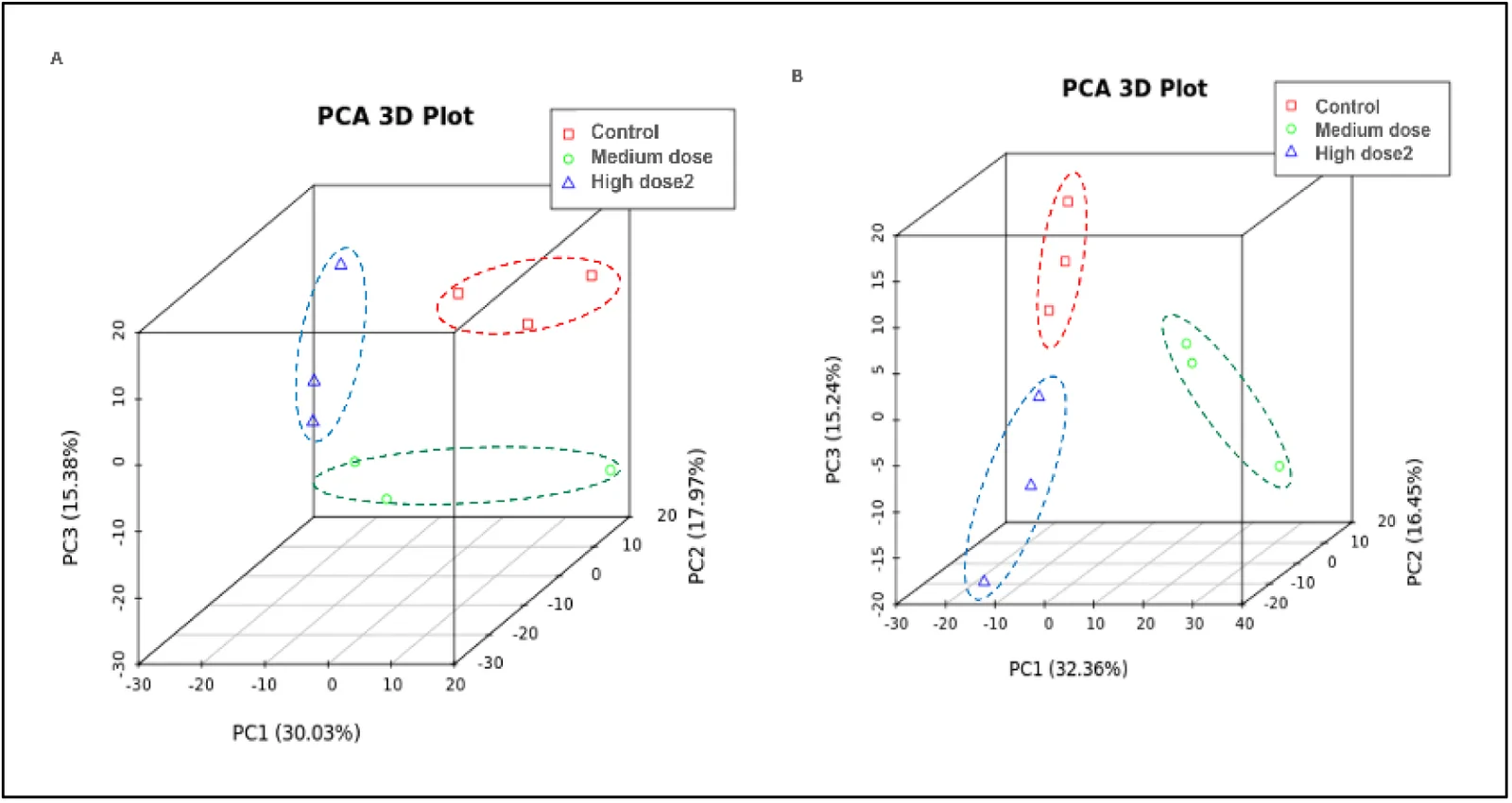
Principal component analysis (PCA) of samples in the indicated groups for lung (A) and heart tissues (B) (*n* = 3 per group).

**Figure 8:**
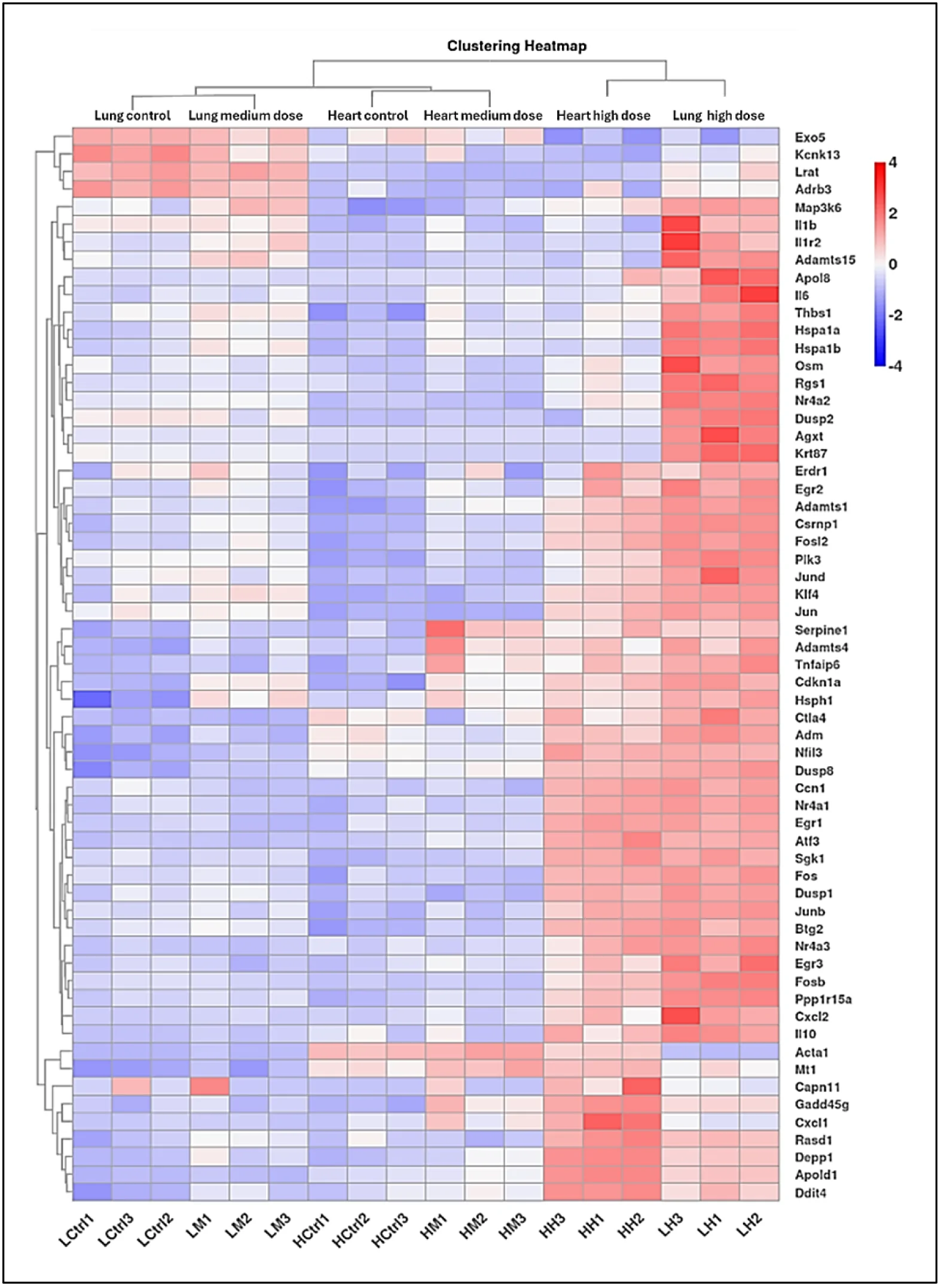
Heatmap of the top 60 DEGs in the lung and heart tissues of the control (unexposed) group and groups exposed to medium and high2 PH_3_ doses, illustrating clustering of samples and gene expression patterns in response to PH_3_ exposure.

The differential analysis of lung tissues showed a dose-dependent increase in the differentially expressed genes (DEGs) after PH_3_ exposure. There were 193 and 814 DEGs identified in the lung tissue exposed to medium and high2 PH_3_ doses, respectively (Figure 9A, Figure 10A), compared to the control group, while 660 and 259 DEGs were identified in the heart tissue of the same exposed groups (Figure 9D, Figure 10D) using a threshold of 0.05 for statistical significance (padj) and a log 2-fold change (Log_2_FC) of expression with an absolute value of at least 1. The enrichment analysis of DEGs in lung and heart tissues revealed that the majority of upregulated genes included those involved in the critical cellular processes such as protein synthesis (e.g., transcription chaperones—*Hspa1a* and *Hspa1b*); cell cycle (*Cdkn1a, ATF3, Csrnp1*); transcription factors and regulators (*Fos, Fosb, Fosl2, Jun, Junb, ATF3, Nr4a3, Egr1, Egr2*); related to extracellular matrix (ECM) and connective tissues (*Adamts1, Adamts4*); inflammatory response (*Cxcl1, Cxcl2, Ccl4, Ccl17, Ptgs2*); and stress response (*Hspa1a and b, Cdkn1a, Cxcl2, ATF3, Sgk1, Gadd45g, Ddit4*), as shown in Table S2 and Figure 8.

**Figure 9:**
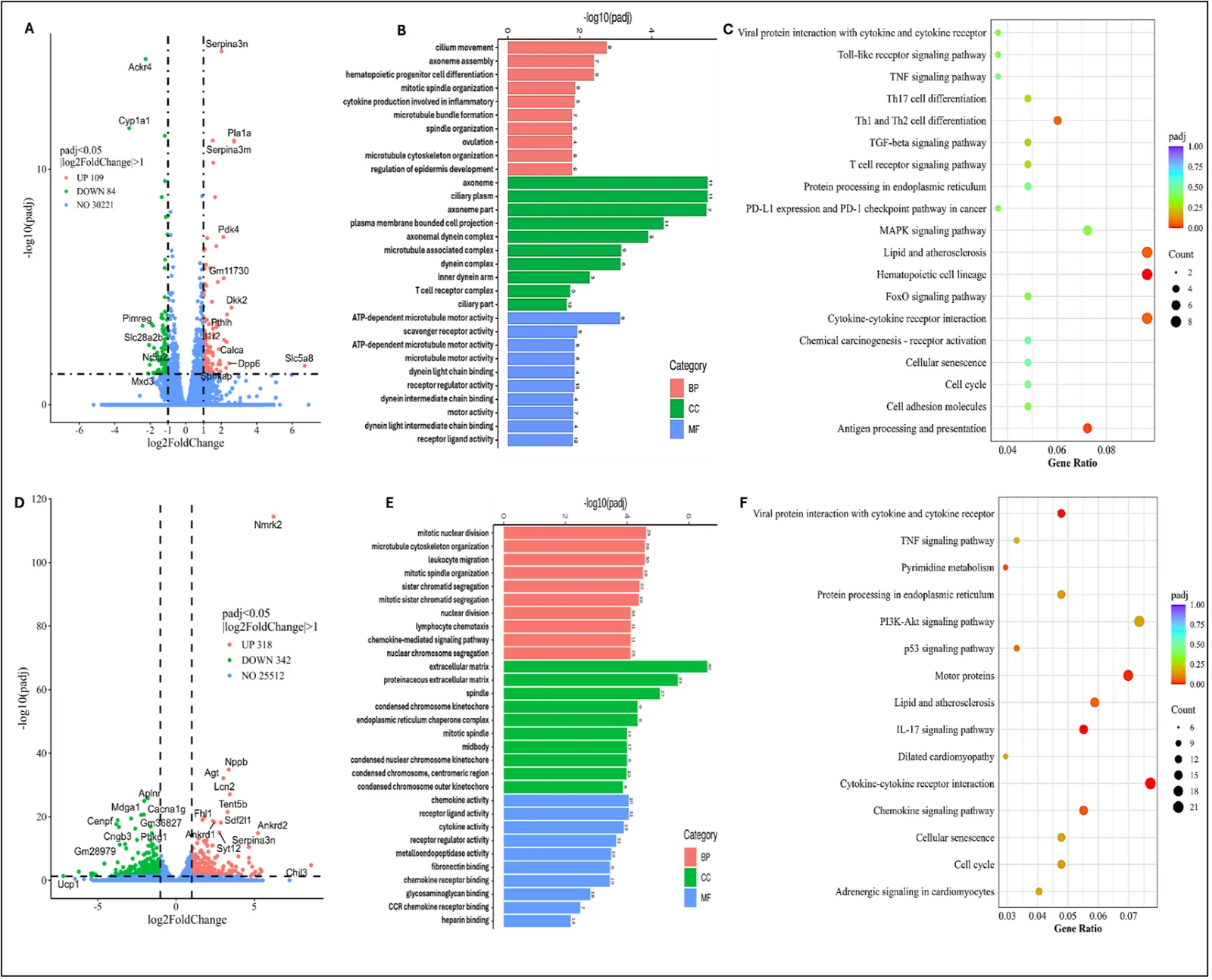
Volcano plot of differentially expressed genes (DEGs) after exposure to the medium PH_3_ dose in the lung (A) and heart (D). All DEGs are represented as a log 2-fold change (*x*-axis) and the significance of the change (*y*-axis). The significance is represented as the negative log (base 10) of the adjusted *p*-value (padj). The dotted lines represent the thresholds used to select the DEGs: 1 for expression change and 0.05 for significance. Upregulated genes (positive log fold change) are shown in red, while downregulated genes are shown in green. Bar plot of enriched Gene Ontology (GO) of lung tissue (B) and heart (E) exposed to the medium dose (*n* = 3 per group). The plot shows the −log10 of adjusted *p*-value on *y*-axis and the enriched pathways on *x*-axis. BP: Biological Process. MF: Molecular Function. CC: Cellular Component. Dot plot of enriched Kyoto Encyclopedia of Genes and Genomes (KEGG) of lung (C) and heart (F) exposed to medium PH_3_ doses. The plot shows the enriched pathways on *x*-axis and the gene ratio on the *y*-axis.

**Figure 10:**
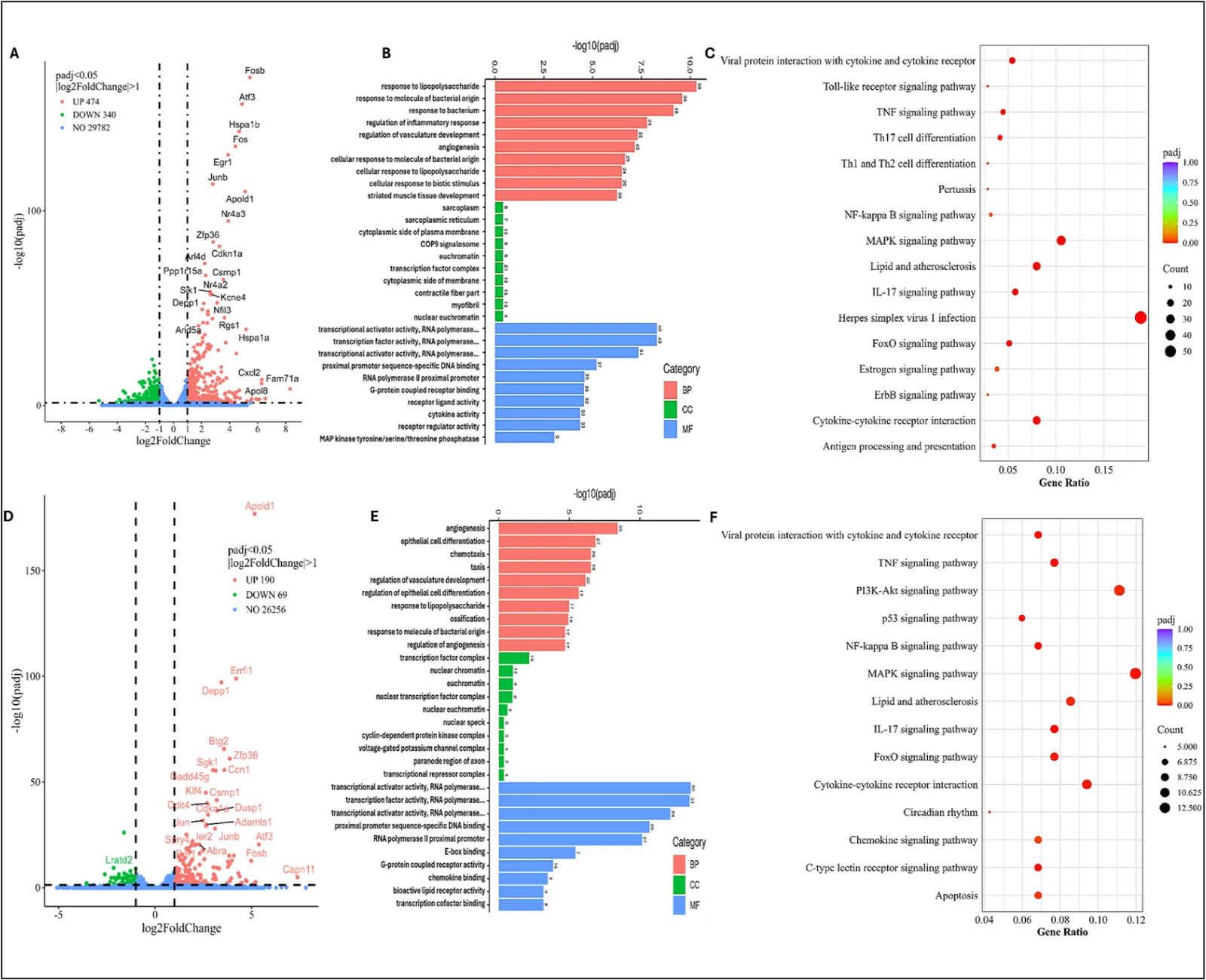
Volcano plot of differentially expressed genes (DEGs) after exposure to high2 PH_3_ dose in lung (A) and heart (D). All DEGs are represented as the log 2-fold change (*x*-axis) and the significance of the change (*y*-axis). The significance is represented as the negative log (base 10) of the adjusted *p*-value (padj). The dotted lines represent the thresholds used to select DEGs: 1 for expression change and 0.05 for significance. Upregulated genes (positive log fold change) are shown in red, while downregulated genes are shown in green. Bar plot of enriched Gene Ontology (GO) of lung tissue (B) and heart (E) exposed to the high2 dose (*n* = 3 per group). The plot shows the −log10 of the adjusted *p*-value on *y*-axis and the enriched pathways on *x*-axis. BP: Biological Process. MF: Molecular Function. CC: Cellular Component. Dot plot of enriched Kyoto Encyclopedia of Genes and Genomes (KEGG) of the lung (C) and heart (F) exposed to the high2 PH_3_ doses. The plot shows the enriched pathways on *x*-axis and the gene ratio on the *y*-axis.

Furthermore, GO pathway analysis demonstrated that the most enriched GO biological processes of DEGs in the lung tissues exposed to medium doses of PH_3_ were related to microtubule organization and mitotic spindle assembly, alongside processes related to cilium assembly and movement (Figure 9B, Table S3). These findings suggest that medium-level PH_3_ exposure disturbs the cytoskeletal dynamics in pulmonary cells—possibly reflecting damage to ciliated airway epithelial cells and activation of cell-cycle pathways for repair. In addition, the most enriched GO biological processes of DEGs in the lung exposed to High2 were related to the immune response to microbe-associated molecular patterns such as lipopolysaccharide, and regulation of inflammatory responses and angiogenesis (Figure 10B, Table S4). These enriched terms suggest that PH_3_-injured lungs mount an acute innate immune response akin to that seen during Gram-negative bacterial infection.^42^ Correspondingly, multiple cytokine and chemokine genes were upregulated, driving processes such as leukocyte recruitment and activation in lung tissue, which align with neutrophilic aggregates shown in the histopathological sections of the lung after exposure. This acute inflammatory storm also contributes to pulmonary thrombus formation, as evidenced by platelet aggregates (platelet thrombi) with neutrophils. Taken together, the lung transcriptomic profile demonstrates a clear dose-dependent shift: medium-dose PH_3_ primarily perturbs cytoskeletal and cell-cycle processes, whereas high2 dose elicits broad inflammation, stress, and proangiogenic pathways in pulmonary tissue. Furthermore, the GO enrichment profile for the heart exposed to medium doses of PH_3_ suggests a dual response involving both cell-proliferation machinery and immune signaling (Figure 9E, Table S5). On the one hand, there was strong enrichment of cell-cycle and mitotic processes: e.g., nuclear division and chromosome segregation were highly significant categories in the PH_3_ exposed group. These suggest that PH_3_ exposure induced cardiac injury that promoted cell-cycle reentry or proliferation signals in certain cardiac cell populations, which are essential for initial wound healing. On the other hand, immune and inflammatory pathways were clearly upregulated; GO analysis identified significant enrichment of leukocyte migration processes, indicating that chemotactic signals (e.g., chemokines) are elevated to recruit immune cells into the heart.^43^ Consistently, several cytokine and chemokine genes were induced, with numerous inflammatory mediators (interleukins, chemokines) showing increased expression. This indicates that even modest PH_3_ inhalation triggers a cardiac innate-immune response.

The total number of DEGs in the heart of mice exposed to the high2 dose was in fact lower than that observed when mice were exposed to a medium dose, likely due to the acute toxicity and shorter survival duration at the higher dose (limiting the window for gene induction). Notably, angiogenesis and vascular development emerged as dominant enriched themes in the heart at the high2 dose (Figure 10E, Table S6). Angiogenesis was one of the most significantly enriched biological processes, accompanied by related terms such as blood vessel development and vasculature development. Genes promoting new vessel growth or vascular remodeling were thus strongly upregulated, consistent with the heart responding to PH_3_-induced hypoxia or tissue damage by attempting to increase blood supply and repair.^44^ The heart exposed to a high2 dose of PH_3_ also showed continued immune-related signaling, while chemotaxis and cell migration remained enriched, indicating ongoing recruitment of inflammatory cells. Coupled with the angiogenic and developmental signals, this suggests that the heart is entering an emergency repair or remodeling mode under high-dose PH_3_ exposure.

The KEGG pathway analysis showed that mice exposed to medium PH_3_ doses did not notably impact many pathways in either lung or heart tissues,^29^ suggesting that only minor changes occurred in both tissues (Figure 9C and F, Tables S7, S9). This could help explain the higher survival rates in this group. However, approximately 30 pathways were found to be significantly impacted in the lung and heart of the mice exposed to high2, and the DEGs were strongly correlated with mitogen-activated protein kinase (MAPK) signaling, IL-17 pathway, atherosclerosis, cytokine–cytokine receptor interaction, and TNF signaling pathways (Figure 10C and F, Tables S8, S10). This points to a correlation between these pathways and the fatal effects after exposure in this group.

For instance, the MAPK pathway was one of the most impacted pathways in both the lung and heart tissues after PH_3_ exposure. The MAPK cascade consists of serine threonine kinases and is classified into three major subgroups: extracellular signal-regulated kinases (ERK), *c-Jun* N-terminal kinases (JNK), and p38 MAPKs. Activation of these kinases occurs through a hierarchical phosphorylation cascade initiated by MAPK kinase kinases (*MAP3Ks*). MAPK signaling pathways regulate a wide range of cellular processes involved in both cell survival and cell death, including cell proliferation, differentiation, migration, and stress responses.^45^ Prolonged or dysregulated MAPK activation can lead to aberrant gene expression, uncontrolled cell proliferation, and apoptosis. MAPK pathways are negatively regulated through dephosphorylation by protein phosphatases, particularly dual-specificity phosphatases (*DUSPs*), which deactivate MAPKs by targeting key regulatory residues. Notably, stimuli that activate MAPKs, including environmental stress and growth factor signaling, also induce *DUSP* expression, highlighting a tightly regulated feedback mechanism.^45^ The transcription factors *Fos*, *Jun*, and *ATF* (*ATF2*, *ATF3*) (early response proto-oncogenes) are key parts of the JNK group that form dimers known as the activator protein-1 (AP-1) complex. *Fos* proteins heterodimerize with *Jun*, whereas *Jun* proteins are also capable of forming homodimeric AP-1 complexes. AP-1 family members act as environmental biosensors, responding to various external toxic stimuli and regulating gene expression involved in many biological processes, including epithelial injury, cellular proliferation, differentiation, and apoptosis.^45–47^ Previous studies indicated the crucial role that MAPK plays under stress conditions, including increased ROS levels and hypoxia, as it converts stress signals into cellular responses, which promote transcription of various inflammatory and apoptosis genes, leading to progression of several diseases.^48,49^ In our study, we found that there was a significant upregulation of the transcription factors *Fos*, *Fosb*, *Jun*, and *Junb* in the lung and heart tissues following PH_3_ exposure (Table S2). This finding is consistent with previous work demonstrating upregulation of *Fos* in the cardiomyocyte following PH_3_ exposure.^29^ In addition, there was upregulation of *DUSP* genes as a feedback mechanism to control the activation of MAPKs. Overexpression of *Fos*/*Jun* (AP-1) has been noted in various cancers,^50,51^ inflammatory diseases,^48,52^ fibrosis,^53^ ventilator-induced acute lung injury,^54^ and other pathological lung conditions. The transcriptional activity of AP-1 is redox-sensitive through the conserved cysteine residues located in the DNA-binding domain. Therefore, the ROS generated after PH_3_ exposure can induce AP-1 activity through activation of MAPKs or directly through its cysteine residues. Consequently, AP-1 controls the expression of inflammatory cytokines and apoptosis genes by binding to AP-1 motifs in the promoters of these genes, leading to inflammation and apoptosis.^55^ On the basis of our results obtained from the transcriptomic analysis, we propose the AP-1 complex as a potential key element in inducing injuries after PH_3_ exposure, pinpointing AP-1 as a potential therapeutic target for the treatment of PH_3_-induced toxicity in a clinical setting.

In addition to the AP-1 complex, the nuclear receptor subfamily 4, group A (*Nr4a1*, *Nr4a2*, *Nr4a3*), which comprises intracellular transcription factors, plays a part in the cellular stress response, cellular differentiation, and apoptosis, and their role in many pathological conditions, including inflammation and oxidative stress-related conditions in lung^56^ and heart tissue^57,58^ have been well studied. *Nr4a1* translocates from the nucleus to mitochondria under conditions of cellular stress, leading to apoptosis, and knocking out *Nr4a1* has been shown to alleviate inflammation, oxidative stress, and apoptosis in an LPS-induced acute lung injury model.^56^ Taken together, the overexpression of AP-1 and *Nr4a* after PH_3_ exposure indicates their potential roles in inducing the tissue damage observed subsequent to exposure.

Exposure to High2 resulted in a significant upregulation of genes involved in cell-cycle regulation and DNA repair, including *cyclin-dependent kinase inhibitor 1A* (*Cdkn1a*), *Gadd45*, and *Ddit4*. *Cdkn1a* encodes P21 protein, which plays a role in cell-cycle arrest by inhibiting cyclin-dependent kinase activity. p21 protein expression has been used as a biomarker for cellular responses to toxic stimuli and DNA damage, and it has been shown to be increased in patients with chronic obstructive pulmonary disease and idiopathic pulmonary fibrosis.^59^ In addition, p21 contributes to several crucial pathways, including DNA replication and repair, transcription, and programmed cell death.^60^ The significant upregulation of *Cdkn1a* in lung and heart tissue after PH_3_ exposure indicates that DNA damage occurred after exposure and can be considered an attempt by the cells to mitigate the harmful effects of the exposure by initiating cell-cycle arrest, allowing the cells time to repair DNA damage and maintain genomic stability. However, as stressful conditions persist and because of the severe damage that occurred after High2 exposure, the cells would likely initiate apoptosis. In addition, *Gadd45g* mRNA was upregulated after High2 exposure. *Gadd45* is a conserved family of stress-response regulators that play critical roles in maintaining genomic stability. *Gadd45* expression is rapidly induced by cellular stresses such as DNA damage and oxidative stress and modulates key processes including cell-cycle arrest, DNA repair, apoptosis, and senescence, through interactions with cell-cycle regulators, DNA repair machinery, and stress-activated kinases.^61,62^ Furthermore, *Ddit4* plays a key role in cellular proliferation and differentiation. *Ddit4* expression is very low in normal lung cells; however, its expression is significantly upregulated under stress conditions like DNA damage, oxidative stress, and hypoxic conditions. *Ddit4* encodes REDD1, a stress-responsive protein known to play a pivotal role in cigarette smoke-induced alveolar injury, increasing inflammation and promoting alveolar cell apoptosis by inhibiting mTOR.^63,64^ Following high2 exposure, *Ddit4* was significantly overexpressed, reflecting the oxidative stress and DNA damage triggered by PH_3_ exposure and highlighting its likely contribution to the resulting lung injury.

### 3.9 Strengths, Limitations, and Future Directions

This study investigated pulmonary toxicity induced by acute inhalation exposure to PH_3_ gas by using a whole-body inhalation exposure model. The findings provide a comprehensive transcriptomic characterization of potential molecular responses to exposure and identify potential mechanisms underlying lung and heart tissue injury following exposure. However, several limitations must be acknowledged. First, only female mice were included in the study, which limits the generalizability of the findings. Future studies incorporating both sexes are needed to determine whether sex-specific differences exist in molecular signaling pathways, including the AP-1 response following exposure, and to assess whether the hormonal status may influence the magnitude of these responses. The main focus of the study was the pulmonary system; comprehensive assessment of extrapulmonary effects, including functional cardiovascular responses, was beyond the scope of our current study. Transcriptomic analysis revealed significant upregulation of genes involved in critical cellular processes, and our results suggest that the AP-1 complex may play a key role in inducing tissue injuries following high-dose PH_3_ exposure. Nevertheless, this study did not include any functional validation of the most significantly altered genes or an evaluation of JNK/AP-1 pathway-modulating molecules. Therefore, additional studies are required to validate these findings and to investigate the therapeutic potential of AP-1-modulating molecules for the treatment of acute PH_3_ toxicity.

## 4 Conclusion

This research represents a comprehensive analysis of the potential molecular mechanisms underlying lung and heart tissue injuries following PH_3_ exposure using a whole-body, unrestrained, and unanesthetized mouse model. Exposure to low or medium PH_3_ doses showed mild changes in respiratory dynamics and histopathology. Inhalation exposure to high doses of PH_3_ induced marked changes in clinical observations, respiratory dynamics, histopathology, and gene expression, highlighting the lethal effects on the pulmonary and cardiovascular systems. During exposure to high doses of PH_3_, mice became lethargic, exhibited piloerection and hunched posture, and some of them experienced slight tremors. They also experienced a marked decrease in respiratory rate and minute volume, leading to euthanasia within 0.5–5 h postexposure, suggesting that severe tissue and systemic damage developed after exposure. Our RNA-seq data from lung and heart tissues showed that medium PH_3_ exposure perturbs cytoskeletal and cell-cycle processes and triggers innate-immune responses. Gene expression analysis after high2 exposure revealed the upregulation of genes involved in critical cellular processes such as protein synthesis, the cell cycle and apoptosis, transcription factors and regulators, inflammatory response genes, and stress response genes. We propose the AP-1 complex as a potential key element in inducing injuries after high2 PH_3_ exposure, implicating AP-1 as a potential therapeutic target in the treatment of PH_3_-induced toxicity in clinical practice.

## Supplementary Material





## Notes

Additional figures and tables including the concentration of PH_3_ gas and the percentage of O_2_ during animal exposures (Figure S1), HBEC cell viability after exposure to different PH_3_ gas concentrations (Figure S2), summary of the quality control results of the RNA samples (Table S1), and the top 35 most significantly upregulated transcripts within the mouse lung and heart tissues following exposure to High2 PH_3_ (Table S2) (PDF)
GO and KEGG significantly altered pathways and associated statistical results in response to PH_3_ exposure (XLSX)

## Abbreviations

PH_3_: phosphine
BALF: bronchoalveolar lavage fluid
WBP: whole body plethysmography
H&E: hematoxylin and eosin
HBECs: human bronchial epithelial cells
ROS: reactive oxygen species
CM-H2DCFDA: chloromethyl-2′,7′-dichlorodihydrofluorescein diacetate
DCF: 2′,7′-dichlorofluorescein
RMFI: relative mean fluorescence intensity
DEGs: differentially expressed genes
Padj: adjusted *p*-value
Log2FC: log 2-fold change
GO: Gene Ontology
KEGG: Kyoto Encyclopedia of Genes and Genomes
RNaseq: mRNA sequencing
PCA: principal component analysis
MAPK: mitogen-activated protein kinase
JNK: c-Jun N-terminal kinases
ERK: extracellular signal-regulated kinases
*DUSP*: dual-specificity phosphatases
AP-1: activator protein-1
*Cdkn1a*: cyclin-dependent kinase inhibitor 1A
