# Supplementary material for: Pulmonary Injury, Functional Impairment, and Transcriptomic Responses Following Phosphine Inhalation in Mice: Implications for Therapeutic Development
Source: Chem Res Toxicol. 2026 Sep 28;39(10):1985–99. doi: 10.1021/acs.chemrestox.6c00223 (PMC13685491; doi:10.1021/acs.chemrestox.6c00223)
Supplement: Supplementary file 1 [file tx6c00223_si_001.pdf]

Supporting Information

Pulmonary Injury, Functional Impairment, and Transcriptomic Responses Following Phosphine Inhalation in Mice: Implications for Therapeutic Development

[1]Esraa Mohamed, [2]Andrea Adamcakova-Dodd*, [2]Xuefang Jing, [3]David K. Meyerholz, [1]Mohammad Al-Natour, [2]Peter S. Thorne*, [1]Aliasger K. Salem*.

[1] Department of Pharmaceutical Sciences and Experimental Therapeutics, College of Pharmacy, University of Iowa, Iowa City, IA, 52242, USA.

[2] Occupational and Environmental Health, College of Public Health, University of Iowa, Iowa City, IA, 52242, USA.

[3] Department of Pathology, Carver College of Medicine, University of Iowa, Iowa City, IA, 52242, USA.

*Corresponding author emails: aliasger-salem@uiowa.edu, peter-thorne@uiowa.edu, andrea-a-dodd@uiowa.edu

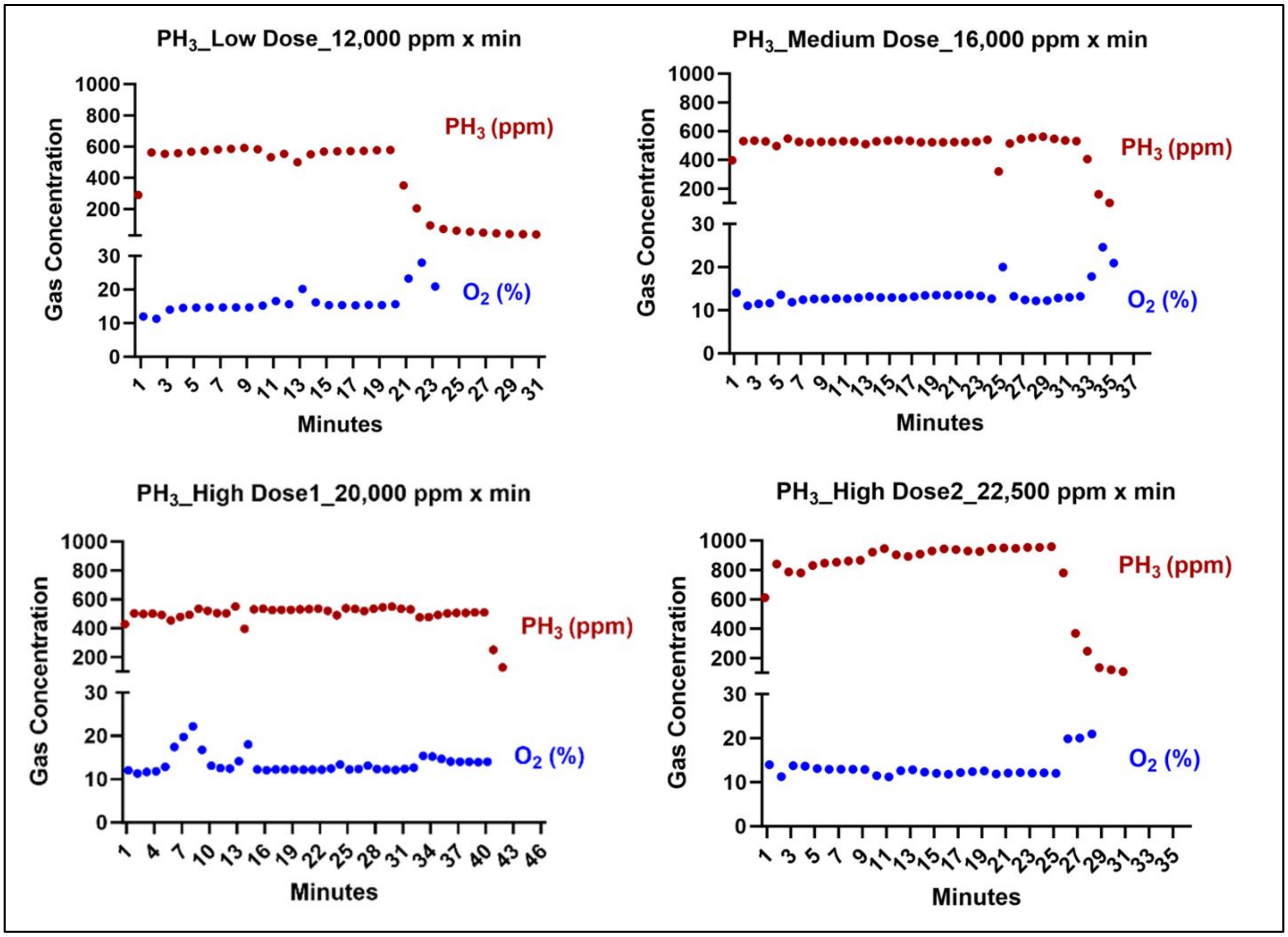

Figure S1: Concentration of PH_3 gas (ppm) and percentage of O_2 measured in real time (using gas monitors) during animal exposures in the whole-body exposure chamber exposed to 4 different PH_3 concentrations for certain time. The total exposure concentrations are products of the PH_3 concentration in the chamber in ppm and the length of the exposure period in mins (ppm x min).

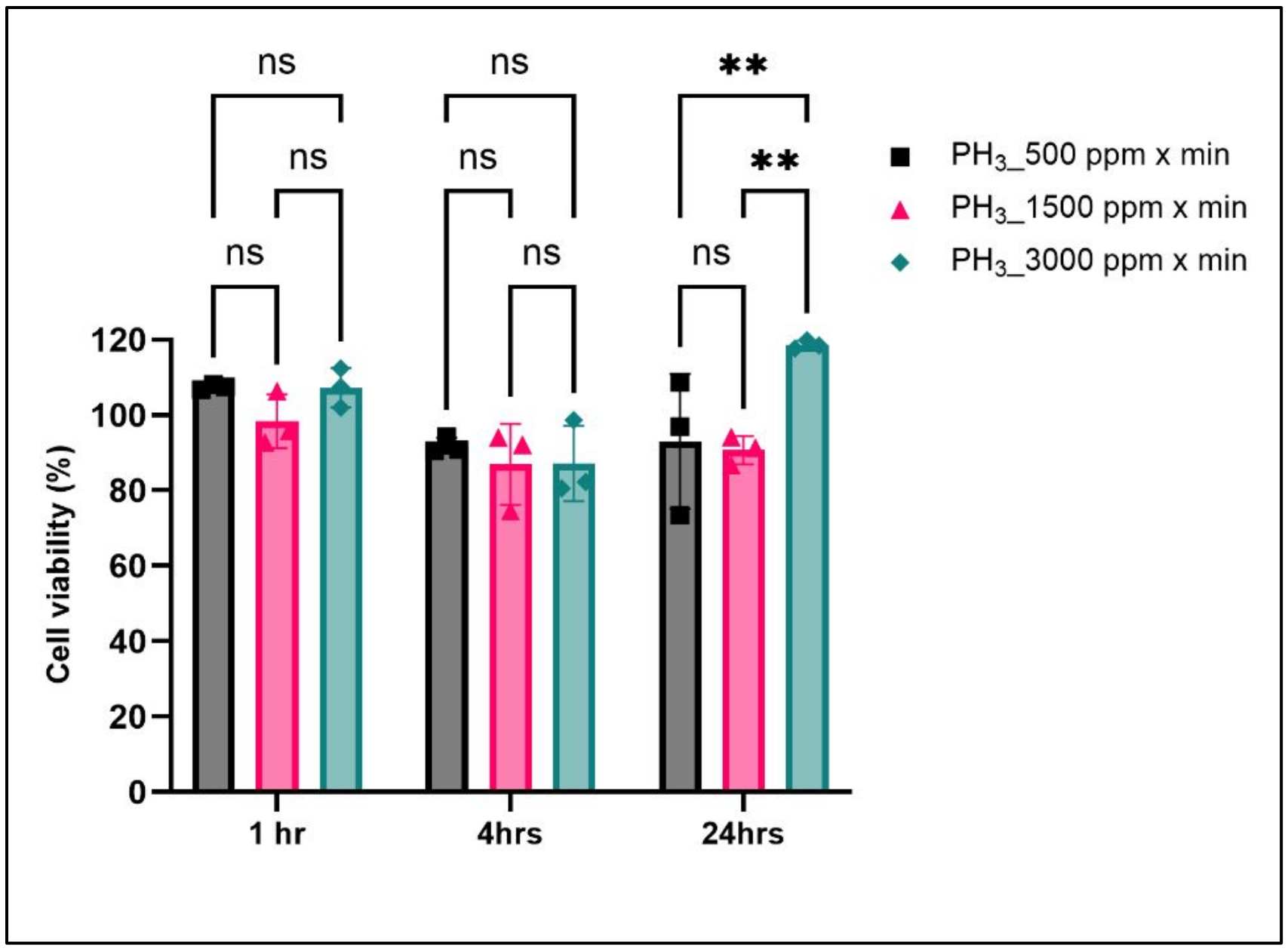

Figure S2: HBECs cell viability measured using alamarBlue after exposure to different PH_3 gas concentrations compared to unexposed cells. The evaluation was performed at 1, 4, and 24 h following exposure (values represent means ± SD). Statistical analysis was performed using two-way ANOVA. *p <0.05, ** p <0.01, ***p <0.001, ****p <0.0001.

Table S1: Summary of the quality control results of the RNA samples prior to sequencing analysis including RNA concentration and integrity values.

No.	Group Name	Sample Name	Concentration (ng/µL)	Volume (µL)	Total Amount (µg)	Integrity value	Sample QC Results
1	Lung_control	LCtrl1	94.52	8.5	0.80342	9.8	Pass
2		LCtrl2	110.89	8.5	0.94257	9	Pass
3		LCtrl3	154.80	8.5	1.31580	9.6	Pass
4	Lung_Medium	LM1	89.34	8	0.71472	9.2	Pass
5		LM2	177.97	8.5	1.51275	9.9	Pass
6		LM3	190.59	8.5	1.62002	9.4	Pass
7	Lung_High2	LH1	149.73	8.5	1.27271	8.6	Pass
8		LH2	144.90	8.5	1.23165	9.3	Pass
9		LH3	257.88	8	2.06304	9.1	Pass
10	Heart_control	HCtrl1	270.50	8	2.16400	9.6	Pass
11		HCtrl2	625.56	8	5.00448	8.6	Pass
12		HCtrl3	245.29	8	1.96232	9.5	Pass
13	Heart_Medium	HM1	207.33	8	1.65864	9.3	Pass
14		HM2	363.31	8	2.90648	9.7	Pass
15		HM3	259.50	8	2.07600	9.7	Pass
16	Heart_High2	HH1	498.84	8	3.99072	8.8	Pass
17		HH2	303.10	8	2.42480	9.5	Pass
18		HH3	278.44	8	2.22752	9.6	Pass

Table S2: Top 35 most significantly up-regulated transcripts within the mouse lung and heart tissues following exposure for High2 PH_3. The table shows the log2-fold change (Log2FC) and adjusted p-values (padj). The genes were ranked based on the Log2 FC in the lung tissue. NS: Non-significantly expressed in that tissue.

Up-regulated DEGs		Lung tissue		Heart tissues	
**Gene ID**	**Gene description**	**log2FC**	**padj**	**log2FC**	**padj**
Cxcl2	Chemokine (C-X-C motif) ligand 2	6.3014	5.24E-14	3.9428	3.56E-05
Apol8	Apolipoprotein L 8	6.2751	6.25E-12	NS	NS
Fosb	FBJ osteosarcoma oncogene B	5.4489	3.00E-169	4.9813	2.22E-13
Hspa1a	Heat shock protein 1A	5.1748	9.12E-40	1.6548	3.15E-05
Apold1	Apolipoprotein L domain containing 1	5.1154	1.42E-110	5.1590	1.49E-177
Atf3	Activating transcription factor 3	4.8902	1.46E-155	5.3715	3.38E-21
Hspa1b	Heat shock protein 1B	4.6881	1.62E-141	NS	NS
Nr4a1	Nuclear receptor subfamily 4, group A, member 1	4.4864	2.20E-27	4.3130	1.32E-07
Fos	FBJ osteosarcoma oncogene	4.4189	7.68E-134	4.3826	4.20E-06
Il1r2	Interleukin 1 receptor, type II	4.3446	6.23E-06	NS	NS
Il6	Interleukin 6	4.2102	5.23E-07	NS	NS
Egr1	Early growth response 1	3.8962	1.91E-129	3.7714	4.41E-13
Osm	Oncostatin M	3.7138	5.81E-07	3.8617	0.00068
Csrnp1	Cysteine-serine-rich nuclear protein 1	3.5524	2.64E-65	3.1864	4.27E-42
Egr3	Early growth response 3	3.5421	1.81E-13	2.2216	6.20E-07
Dusp1	Dual specificity phosphatase 1	3.4095	5.15E-18	3.2134	3.49E-37
Cdkn1a	Cyclin-dependent kinase inhibitor 1A (P21)	3.2794	1.75e-82	2.7469	2.94E-35
Thbs1	Thrombospondin 1	3.2782	2.47E-14	2.4053	0.00734
Rasd1	RAS, dexamethasone-induced 1	3.1940	5.18E-29	3.1544	0.00013
Ccn1	Cellular communication network factor 1	3.1253	6.19E-14	3.5839	2.14E-56
Nfil3	Nuclear factor, interleukin 3, regulated	3.1158	1.84E-53	1.1792	4.44E-07
Mt2	Metallothionein 2	3.0627	0.001	1.7963	0.0109
Junb	Jun B proto-oncogene	2.8039	1.98e-114	3.1059	1.05E-28
Mt1	Metallothionein 1	2.6807	7.93E-11	1.0208	7.32E-07
Egr2	Early growth response 2	2.5736	1.36E-29	2.8444	0.03493
Btg2	BTG anti-proliferation factor 2	2.5185	4.85E-32	3.5735	2.17E-66

Ddit4	DNA-damage-inducible transcript 4	2.5082	4.68E-31	2.7067	1.23E-40
Adamts1	A disintegrin-like and metallopeptidase with thrombospondin type 1 motif, 1	2.4466	6.78E-49	2.6672	1.31E-30
Serpine1	Serine (or cysteine) peptidase inhibitor	2.4096	4.50E-43	1.883	0.00644
Depp1	DEPP1 autophagy regulator	2.1633	4.25E-53	3.4371	7.38E-98
Jun	Jun proto-oncogene	1.6770	5.16E-25	2.4779	1.72E-32
Map3k6	Mitogen-activated protein kinase kinase kinase 6	1.6641	5.01E-18	1.7415	1.27E-11
Gadd45g	Growth arrest and DNA-damage-inducible 45 gamma	1.5150	6.07E-15	3.1467	4.25E-56
Erdr1	Erythroid differentiation regulator 1	1.2458	0.0093	2.1929	1.38E-02
Cxcl1	Chemokine (C-X-C motif) ligand 1	NS	NS	3.9785	1.48E-13
